# Study on the correlation between hTREC and HPV load and cervical CINI/II/III lesions and cervical cancer

**DOI:** 10.1002/jcla.23257

**Published:** 2020-02-26

**Authors:** Haizhen He, Qionghui Pan, Jiajia Pan, Yumei Chen, Liqin Cao

**Affiliations:** ^1^ Wenzhou People's Hospital Gynecotokology Wenzhou China; ^2^ Shanghai TCM‐Integarted Hospitalgynecotokology Shanghai China

**Keywords:** cervical cancer, cervical precancerous lesions, hTREC, human papillomavirus

## Abstract

**Objective:**

To investigate the correlation between hTREC and human papillomavirus (HPV) load and cervical intraepithelial neoplasia (CIN) grade II/III lesions and cervical cancer.

**Methods:**

A total of 135 patients with cervical lesions of different degrees admitted to our hospital from January 2016 to February 2017 were selected, including CIN I/III 65 cases, grade III 39 cases, and cervical cancer 31 cases. The expression of hTERC gene was detected by fluorescence in situ hybridization (FISH) in three groups, and the HPV load was detected by second‐generation hybridization capture (HC II) method, and its relationship with cervical lesion grade was analyzed. Department.

**Results:**

The positive expression rate of hTERC gene amplification was cervical cancer > CIN I/II lesion > CIN III lesion; the positive expression rate of HPV was cervical cancer > CIN I/II lesion > CIN III lesion. After treatment, the positive rate of hTERC gene amplification and HPV expression decreased significantly within 1 year (*P* < .05). Spearman's analysis showed that the degree of cervical lesion was positively correlated with hTREC and HPV load (*P* < .05).

**Conclusion:**

hTREC and HPV are closely related to the occurrence and development of cervical precancerous lesions and cervical cancer. The abnormal amplification of hTERC gene increases with the grade of cervical lesions. Both of them can be used as auxiliary indicators for early screening, treatment, and prognosis of cervical cancer.

## 1 INTRODUCTION

Cervical cancer is one of the most common malignant tumors in gynecology, second only to breast cancer, and its morbidity and mortality rate ranked second, and showed a gradually increasing and younger trend.^1^ Early diagnosis and effective prognosis of cervical cancer are of great significance for patients' long‐term survival, especially for the early detection, early treatment of cervical intraepithelial neoplasia (CIN), as well as the improvement of its screening accuracy, are particularly important for the prevention and treatment of cervical cancer.^2^, ^3^ Relevant research confirms that human papilloma virus (HPV) can greatly increase the incidence of cervical cancer.^4^ When HPV continues to infect the cervical area and invade the mucosal squamous epithelial cells, which will gradually develop lesions from CIN I, CIN II, and CIN III, and eventually, the cancer cells will break through the basement membrane and develop into invasive cervical cancer. Therefore, people should attach great importance to it. Many studies have shown that telomerase is strongly related to cell canceration. HTERC is the main part of telomerase, and its expansion will cause cervical cells to be very prone to atypical development, and then evolve into cervical cancer.^5^ In this study, hTERC gene detection was performed by fluorescence in situ hybridization (FISH) in patients with different cervical CIN lesions, and the hybrid capture two generation test (HC2) was used to detect their HPV load, which were analyzed in order to provide evidence for the diagnosis and treatment of cervical cancer and precancerous lesions.

## 2 MATERIALS AND METHODS

### 2.1 General information

A total of 135 patients with cervical lesions of different degrees in our hospital from 2016.1 to 2017.2 were selected as the research objects. Inclusion criteria: (a) Patients with cervical precancerous lesions and cervical cancer diagnosed pathologically and newly treated in our hospital; (b) Case data are complete and the survival time after treatment is greater than or equal to 1 year; (c) Sign the informed consent. Exclusion criteria: (a) Patients with other malignant tumors; (b) Patients whose cervical cancer were failed to control or relapse after previous treatment. According to the pathological diagnosis results of surgical resection specimens and biopsy specimens, all cases were divided into CIN I/II lesion group (65 cases), CINI III lesion group (39 cases), and cervical cancer group (31 cases). The general information of the three groups was not obvious Difference (*P* > .05).

### 2.2 Method

#### 2.2.1 hTERC gene expression detection

All patients had intercourse banned 1‐2 days before the test. We had the patient's cervix fully exposed using a speculum and slowly cleaned the cervical secretions with a dry cotton swab. Then, we rotated the special sampling brush five times clockwise at the cervical canal for 10 seconds to collect the detached cells once. After brushing, we put the sampling brush into a thin layer of liquid‐based cytology examination preservation solution, and then extracted 10 mL, and centrifuge at 10,000 *g* for 10 minutes (centrifugation radius 15 cm). After removing the supernatant, we cultured the cells with 5 mL collagenase B at 37°C for half an hour and then added 5 mL deionized water and pipetted the suspended cells three times for at 37°C for half an hour. Afterward, we slowly added 2 mL of fixative solution for thorough mixing, and we centrifuged at 10,000 *g* for 10 minutes, removed the supernatant, and then added 5 mL of fixative solution for 10 minutes; then, we centrifuged again and fixed the cells with fixative solution, and lately, slowly blew the suspension cells, extracted the liquid from the tube, and prepared the tablet; pre‐dried after natural drying (preheated, rinsed with SSC solution, soaked with hydrochloric acid solution, fixation with CH_2_O solution, dehydration), we hybridized with the denatured probe overnight at 42°C. After washing the slide, we added 10 μL of DAPI counterstain and determined the gene expression level with a fluorescence microscope.

#### 2.2.2 HPV‐DNA viral load detection

The HC2 method was used to detect the HPV viral load. A sampling brush was used to rotate the patient's cervix counterclockwise three times, and the sample was taken after 10 seconds. The sampling brush was placed in a test tube, and the double‐stranded DNA was broken down into single strands by lysis. The HPV probe solution hybridizes the lysed specimen, captures the hybrids, and amplifies the signal, and then uses a chemiluminescence meter (Digene American Corporation) for interpretation.

#### 2.2.3 Treatment

All patients were given comprehensive radiotherapy, external wide field irradiation, the upper limit of the field was located between the level of the 4th or 5th lumbar vertebra, the lower limit was about 5 cm below the upper margin of the pubic symphysis, and the left and right boundary was 0.5 cm away from the lateral midline of the femoral head, before and after 6MV‐X‐ray irradiation, 200 cGy/1f, five times per week. When the dose DT reached 3000 cGy, the lead plate was used to block the middle of the pelvic cavity, and the irradiation continued. DT was added to 2500 cGy/13f for six times.

### 2.3 Evaluation criteria

#### 2.3.1 Determination of positive hTERC gene expression

Observe the FISH results of cervical exfoliated cells under a 100× objective lens and use red and green filters to record TERC and CSP3 signals. When the red signal value is >2 and the green signal is ≥2, which is indicated as a cell abnormality. Calculate 100 fully signaled cells and record the number of abnormal cells. When the average number of abnormal cells ± 3 standard deviations) × 100% value >7%, hTERC gene amplification is positive; otherwise, it is negative.

#### 2.3.2 The HC2 method was used to detect the HPV load of patients

The HPV load was expressed according to the ratio (RLU/CO) of the relative fluorescence photometric value (RLU) and the HPV positive control domain value (CO) provided by the American Digene company. Positive: RLU/CO＞1; otherwise, the result is negative.

### 2.4 Statistical processing

Data were processed using SPSS19.0 (SPSS Inc) software. Measurement data were expressed as mean ± standard deviation by using t test; count data were expressed as rate, using *χ* ^2^ test; The correlation was analyzed by Spearman. The difference was statistically significant at *P* < .05.

## 3 RESULTS

### 3.1 The results of hTERC gene amplification in patients with different grades of cervical lesions

**Figure 1:**
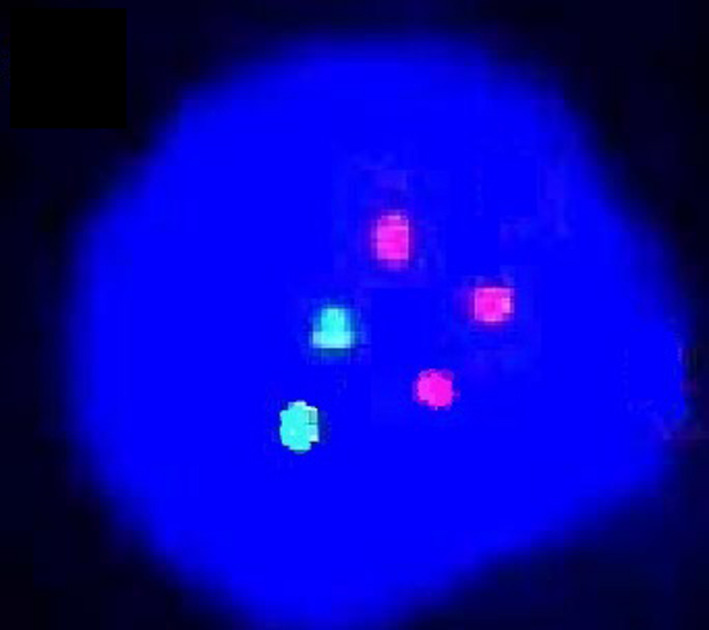
FISH staining results of hTERC gene in patients with CIN I/II lesions (100×)

**Figure 2:**
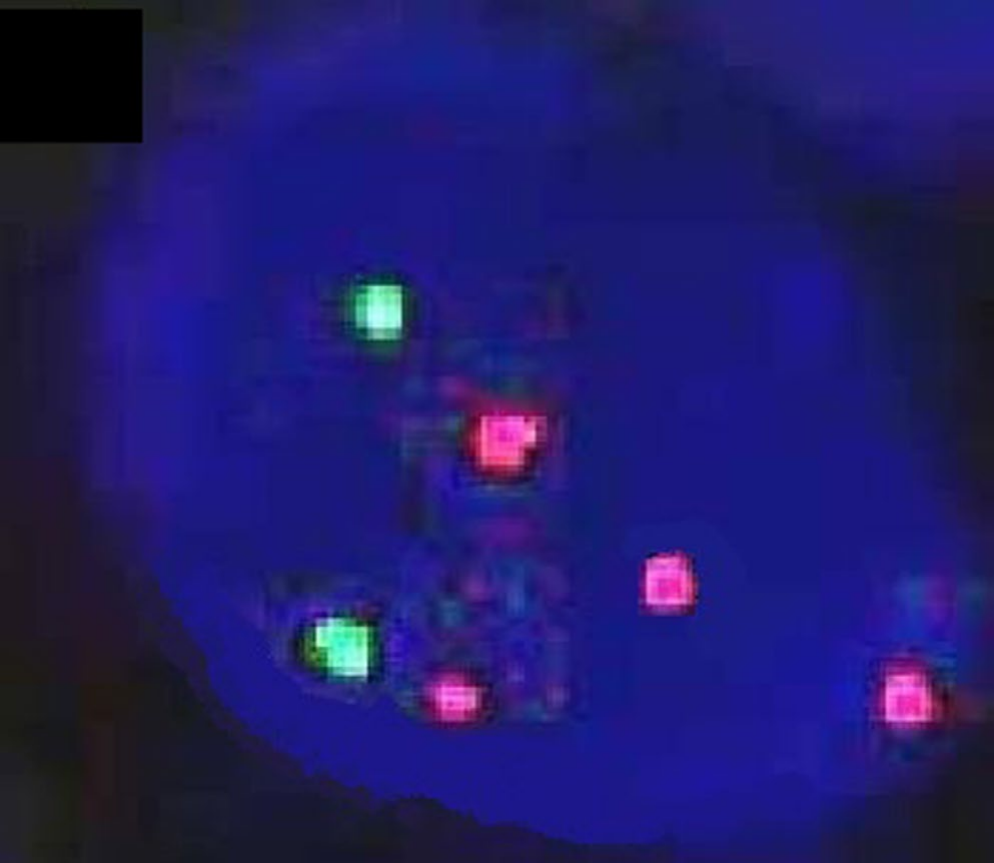
FISH staining results of hTERC gene in patients with CIN III lesions (100×)

**Figure 3:**
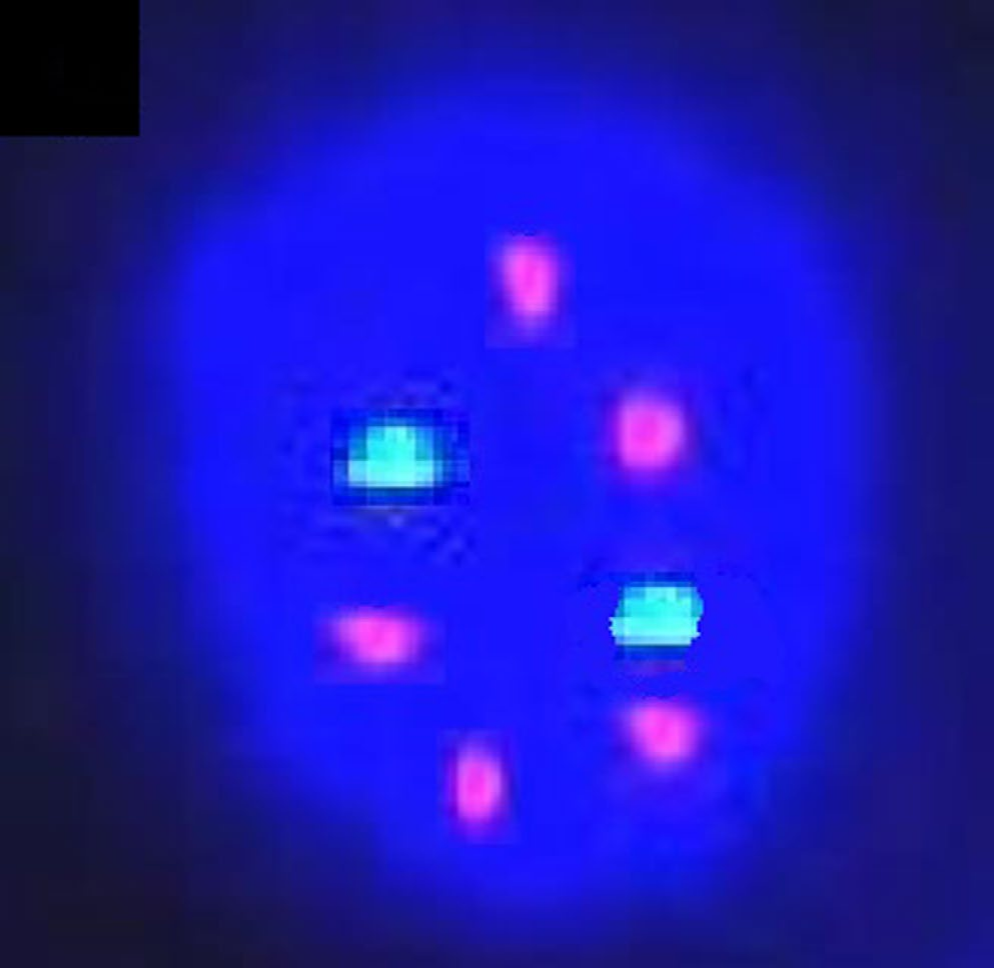
FISH staining results of hTERC gene in cervical cancer patients (100×)

The positive expression rate of hTERC gene amplification is ranked from high to low as manifested as cervical cancer > CIN III lesions > CIN I/II lesions (*P* < .05), as shown in Table 1. From the positive rate of amplification, it can be concluded that the positive rate of hTERC will increase significantly with the severity of cervical lesions. The results of hTERC gene amplification in different cervical lesions are shown in Figures 1, 2, 3.

**Table 1:** Positive rate of hTERC gene amplification in patients with different cervical lesions

Group	Cases	HPV positive cases	HPV positive rate (%)
CIN I/II lesion group	65	41	63.08
CIN III lesion group	39	37	94.87
Cervical cancer group	31	31	100.00
*χ^2^*	25.445		
*P*	＜.001		

### 3.2 Results of HPV load in patients with different levels of cervical disease

The HPV positive rate is ranked from high to low: cervical cancer > CIN III lesions > CIN I/II lesions (*P* < .05). It can be seen that as the severity of cervical lesions increases, the HPV load is also increasing in Table 2.

**Table 2:** HPV positive rates in patients with different grades of cervical lesions

Group	Cases	hTERC gene amplification positive cases	hTERC gene amplification positive rate (%)
CIN I/II lesion group	65	41	63.08
CIN III lesion group	39	37	94.87
Cervical cancer group	31	31	100.00
*χ^2^*	25.445		
*P*	＜.001		

### 3.3 Patients with different grades of cervical lesions before and after treatment

After active treatment, the positive expression rate of hTERC gene amplification and HPV positive rate in different groups of patients decreased significantly within one year (*P* < .05), as shown in Tables 3 and 4.

**Table 3:** Positive expression rate of hTERC gene amplification in patients with different cervical lesions before and after treatment (n, %)

Items	Time	CIN I/II lesion group	CIN III lesion group	Cervical cancer group
hTERC gene amplification positive rate	Before treatment	41 (63.08)	37 (94.87)	31 (100.00)
	After treatment	14 (21.54)	16 (41.03)	18 (58.06)
*χ^2^*		21.304	23.547	14.016
*P*		＜.001	＜.001	＜.001

**Table 4:** HPV positive rate of patients with different cervical lesions before and after treatment (n, %)

Items	Time	CIN I/II lesion group	CIN III lesion group	Cervical cancer group
HPV positive rate	Before treatment	32 (49.23)	35 (89.74)	30 (96.77)
	After treatment	5 (7.69)	11 (28.13)	12 (38.71)
*χ^2^*		25.539	28.031	21.331
*P*		＜.001	＜.001	＜.001

### 3.4 The correlation between the hTREC

Human papillomavirus load, and the degree of cervical lesions, Spearman's correlation analysis results: There was a positive correlation between the degree of cervical lesions and hTREC and HPV load (*r* = .436, 0.385, *P* < .05). As shown in Table 5.

**Table 5:** Spearman's correlation analysis results of hTREC, HPV load, and cervical lesions

Item	*r*	*P*
hTREC gene amplification level	.436	.012
HPV load	.385	.023

## 4 DISCUSSION

Cervical cancer is one of the most common gynecological malignancies that ranks second in the incidence of gynecological malignancies. Besides, cervical cancer can be divided into two types, squamous cell carcinoma, and adenocarcinoma. The former is the most common, accounting for about three‐quarters of cervical cancer. The latter is highly malignant, patients are prone to metastasis, the prognosis is extremely poor, and the mortality rate is high.^6^, ^7^ Middle‐aged and elderly women are a high‐incidence group, but their incidence tends to be younger in recent years, posing a serious threat to women's health, and has been raised to the height of a public health problem.^8^ Relevant statistics show that approximately 480 000 women worldwide are diagnosed with cervical cancer each year.^9^ The 5‐year survival rate of patients is about 70%. If they can be diagnosed early, their 5‐year survival rate can reach 80% or more. Cervical cancer is caused by many factors, including viral infection, sexual behavior, biological factors, and the number of births. Clinical symptoms of early patients are not obvious, and they are easily missed or misdiagnosed clinically.^10^ Cervical cancer is a long‐term pathological process, and its early lesions are reversible.^11^ Therefore, improving the early diagnosis rate and screening for precancerous lesions are of great significance for the prevention and treatment of cervical cancer.

Human papillomavirus is a group of circular double‐stranded DNA viruses. It is small and nonenveloped and widely exists in nature that can cause abnormal proliferation of human skin and mucous membranes, causing papilloma and wart‐like lesions in host tissues.^12^, ^13^ Relevant research shows that the incidence of cervical cancer is significantly correlated with high‐risk HPV infection, and more than 90% of cervical cancer patients will be associated with high‐risk HPV infection.^14^ After HPV infection, if not controlled in time, it may eventually lead to cervical precancerous lesions, eventually induce cervical cancer, and even infect the next generation through childbirth.^15^ When the human HPV virus is infected, it not only induces the body to produce an anti‐viral immune response, but also escapes the body's immune response, making the infection a persistent feature, which weakens the body's ability to remove viruses and greatly increases the prevalence of cervical cancer.^16^ The results of this study showed that the positive rate of HPV was 96.77% in patients with cervical cancer, 89.74% in patients with CIN III lesions, and 49.23% in patients with CIN I/II lesions, indicating a higher HPV load. The higher the level of cervical lesions, the more likely it is because HPV infection is the primary initiating factor for cervical cancer. After invading mucosal squamous epithelium, it will integrate and replicate with human host cell chromosomes with a sustained high load. Continuous invasion in a free state, gradually developing into precancerous lesions and invasive cancer. HPV can be inhibited through effective treatment, and the expression of initiation factors of early and late protein replication can be suppressed, thereby effectively eliminating HPV infection in patients' cervix.^17^ The results of this study show that after active treatment, HPV positive rates in different groups of patients within 1 year. All of them decreased significantly, thus preventing the precancerous lesions of the cervix.

Telomerase is a reverse transcriptase that maintains telomere length and plays an important role in the process of cell canceration.^18^ Relevant research shows that telomerase is highly expressed in more than 90% of malignant tumors, so telomerase activity can be detected clinically as a judgment of benign and malignant tumors.^19^ Although HPV detection technology is currently very mature, there is some controversy in the medical community about its load level and the degree and progression of cervical lesions. It is generally believed that HPV infection alone cannot cause cancer, and other factors are required.^20^ In recent years, the relationship between telomerase and HPV infection in carcinogenesis has become a new research hotspot. hTERC is the main component of telomerase, and abnormal proliferation of hTERC can be found in cervical cells of patients with cervical cancer.^21^ The results of this study show the positive expression rate of hTERC gene amplification is highest in cervical cancer, moderate in CIN III class lesions, and lowest in CIN I/II class lesion, which indicates the level of hTERC is increased with the evolution from hyperplasia to cancer. With the continuous development of the deterioration of cervical lesions, the positive rate will continue to increase. The expansion and the integration of HPV show consistency, resulting in the malignant cycle of cell proliferation. After active treatment, the positive expression rate of hTERC gene amplification was significantly reduced in cervical cells of different patients. This is because the hTERC gene transcription or translation process can be blocked by treatment, thereby inhibiting telomerase activity and making tumor cells apoptosis to achieve the purpose of treating cancer.

In summary, TERC gene amplification and HPV have a very close relationship with cervical cancer and precancerous lesions, and its detection can help improve the early screening rate of cervical cancer and provide new directions for cervical cancer treatment.
